# Polysaccharides of nutritional interest in jicama (*Pachyrhizus erosus*) during root development

**DOI:** 10.1002/fsn3.2746

**Published:** 2022-01-23

**Authors:** Marcela González‐Vázquez, Georgina Calderón‐Domínguez, Rosalva Mora‐Escobedo, Ma. Paz Salgado‐Cruz, José Honorato Arreguín‐Centeno, Ricardo Monterrubio‐López

**Affiliations:** ^1^ Escuela Nacional de Ciencias Biológicas Instituto Politécnico Nacional Ciudad de México México; ^2^ Consejo Nacional de Ciencia y Tecnología (CONACyT) Ciudad de México México; ^3^ Técnológico Nacional de México/IT de Roque Guanajuato México

**Keywords:** inulin, jicama root development, nutraceutical polysaccharides, pectin, starch

## Abstract

Jicama root applications have focused on their nutraceutical properties without clearly specifying which compounds are related to this effect. Thus, the aim of the present study was to identify the changes in polysaccharides of nutraceutical interest in two commercial jicama roots (YS – Yellow Seed; PS – Purple Seed) during four stages of maturation, focusing on starch, fructooligosaccharides, and pectin (via galacturonic acid), and on their glycemic index, with the goal of determining, if possible, the best cost‐effectiveness between jicama growing stages and nutraceutical effect. Both materials (YS, PS) presented similar growth rates (0.069 and 0.072 cm/day) and final sizes (12.7 ± 1.25, 12.3 ± 1.63 cm). Changes in size were accompanied by changes in protein, fiber, ashes, lipids, and carbohydrates, after 106 or 127 days of growing. It was also found that fructose content was higher than glucose during the maturing stages, possibly because of the hydrolysis of fructooligosaccharides or sucrose for starch production. Concerning inulin, its levels decreased (<6.0%), after the first days (YS: 13.4% ± 0.7%; PS: 8.4% ± 0.2%, 106 days); however, during development, the presence of other fructooligosaccharides was observed (nystose‐YS 106 days 15.8% ± 0.9% and PS‐106 days 18.5% ± 0.1%), while galacturonic acid and native starch levels increased, which must be related to the jicama's low glycemic index found (<25%), and their nutraceutical properties. This work proves the presence of inulin in jicama roots by analytical methods, its dependence on root development and classifies jicama as a low glycemic index food, supporting its nutraceutical character.

## Graphical abstract

Study of Pachyrhizus erosus nutraceutical carbohydrates and glycemic index during root development. Pachyrhizus erosus nutraceutical carbohydrates are related to the state of maturity of the root, being inulin the first to be used. Pachyrhizus erosus root can be classified as a low glycemic index food.

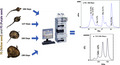

## 1 INTRODUCTION

*Pachyrhizus erosus*, better known as jicama, is a tuberous legume that belongs to the genus *Pachyrhizus*, which is cultivated around the world due to its edible roots, mainly composed of water (87%), starch (10.7%), protein (1.3%) and fiber (1.4%) (Sorensen, 1996), and commonly used as food, cosmetic (Wathoni et al., 2018) or for medical purposes (Lim, 2016a, 2016b).

Jicama root studies have been reported by different authors, citing results about their nutraceutical properties (Buckman et al.,^2018^; González et al., 2018; Khalid & Wan, 2015), including prebiotics (Pulbutr et al., 2021) and dietary fiber (Santoso et al., 2021). For example, a study carried out in murine models regarding the hypoglycemic effect of jicama aqueous extract showed a decrement in blood glucose levels and body weight (Park et al., 2016; Santoso et al., 2019). In other investigations, the role of root phytoestrogens was analyzed by Nurrochmad et al. (2010), who found that they can play a role in the prevention of osteoporosis. Similarly, another study in murine models, about how the insoluble residue of jicama increased the absorption of zinc and iron was carried out by Hayashi et al. (2001). More recently, Baroroh et al. (2021) demonstrated that jicama root extract (soluble fiber) could activate the adaptive immune response by improving the production of cytokines and immunoglobulins (IL‐6, IL‐10, TNF‐a, IgA and IgG) by B cells, while Pulbutr et al. (2021) established that yam bean tuber extract, used as a prebiotic, enhanced the growth of *Lactobacillus L. plantarum*. In 2020 and 2021, Santoso et al. reported that jicama fiber prevents type 2 diabetes mellitus, considering it as a reliable supplement for counteracting the development of this disease. Most of these studies have been carried out in murine models.

Despite the nutraceutical advantages of this root, few works have focused on the specific components that could be related to the aforementioned functional properties (Fernandez et al., 1997), citing polysaccharides, mainly fibers, as the possible source, or just presenting the proximate analysis of the samples, including simple sugars detected by HPLC (Sarkar et al., 2021), and reducing and nonreducing sugar contents (Santoso et al., 2020), without a deeper evaluation to clearly establish what types of polysaccharides are present in the jicama root.

Jicama polysaccharides include starch (Buckman et al., 2018; González et al., 2018; Melo et al., 2003), pectin, cellulose, xyloglucans, heteromannans, heteroxylans (Klockeman et al., 1991; Noman et al., 2007; Ramos de la Peña et al., 2013) and, in much lower quantities, inulin (Stevenson et al., 2007), which could be the fiber material that has been related to the nutraceutical beneficial effects. There are just a few recent studies confirming the presence of fructooligosaccharides: Sarkar et al. (2021) cited its presence in jicama powder, confirmed through its fructose and glucose content. However, we did not find any investigations that present an HPLC methodology for inulin or fructooligosaccharides detection. Jicama roots are consumed at different stages of development, probably having different polysaccharide composition, data that have not been yet reported. Also, this information could be useful for the selection process of raw materials, as the relative amounts of the different types of carbohydrates are dependent on the maturity of the plant tissue (Ramos de la Peña et al., 2013). In this sense, carbohydrate synthesis, including structural and nonstructural components, is related to the root growth process, of which it has been established that tubers are the main sink of carbon produced during photosynthesis, promoting the formation of starch, glucose, fructose, and sucrose (Vaillant & Desfontaines, 1995), in proportions that could result in products with different glycemic indexes. Thus, the aim of the present study was to identify the changes in polysaccharides of nutraceutical interest in two commercial jicama roots (YS – Yellow Seed; PS – Purple Seed) during four stages of maturation, focusing on starch, fructooligosaccharides, and pectin (via galacturonic acid), and on their glycemic index, with the goal of determining, if possible, the best cost‐effectiveness between jicama growing stages and nutraceutical effect.

## 2 MATERIALS AND METHODS

### 2.1 Materials

Two types of jicama root commercial materials (Yellow Seed, YS and Purple Seed, PS) developed up to four different growing stages (106, 127, 160, and 190 days) were harvested (two to four roots per sample) at Apaseo el Grande (2019, Guanajuato, Mexico, 20°29′32.7′′N 100°43′32.5′′W). Jicama tubers were produced following commercial practices: seeds were planted at a density of 30 kg/Ha, one seed at 20 cm intervals; watering 10–12 times depending on the season; no fertilizers were added; manual weeding was done twice during the first three months; chemical deflowering. At the time of sample collection all materials were weighed, washed, and allowed to dry at room temperature (25°C) for 24 hr. These were then cut (MIGSA/AH‐HLC300) into 2 mm‐thick slices and dried (AFOS MINI KLIN) at 30°C for 72 h. Finally, the jicama was ground in an IKA mill (IKA^®^ A11, basic) for 10 min in 30 s lapses and sieved through a 0.5 mm mesh.

### 2.2 Jicama's diameter and growth rate

**FIGURE 1:**
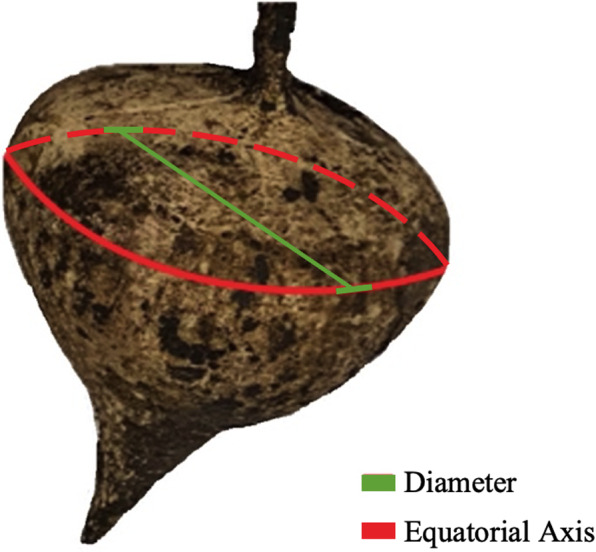
Jicama equatorial axis (diameter)

The growth of the jicama was evaluated by measuring, with a caliper, the diameter of the tuberous roots along their equatorial axis (Figure 1). The growth rate was calculated from the slope of the root diameter versus harvesting time graph. This analysis was performed in the two independent samples (YS, PS) by triplicate, at each harvesting time.

### 2.3 Proximate chemical composition

Moisture was determined on a moisture analyzer (OHAUS MB45) following the manufacturer's methodology. Fresh jicama (1 g) was placed in the thermobalance (108°C, 10 min), directly measuring the humidity percentage of the sample. Ash content was determined according to the AOAC 942.05 methodology while protein content (N × 6.25) was measured with an automated Kjeltec 8200 (FOSS), where 20 mg of sample was weighed, subjected to digestion at 400°C for 30 min, and distilled for 5 min. Titration was carried out with 0.1 N hydrochloric acid (HCl). Lipids were analyzed following the method of Noman et al. (2007) with modifications. The lipids were extracted with petroleum ether at 60°C in a Soxhlet extractor, quantified gravimetrically, and reported as a percentage of lipids.

Neutral detergent fiber (NDF) was determined using a 122 Fibertec™ system (FOSS) following the manufacturer methodology: the sample (0.5 g) was weighed in crucibles to which thermostable α‐amylase was added (100 µl, Sigma‐Aldrich, A3306). The α‐amylase was previously dissolved in 50 ml of neutral detergent solution. This solution was prepared by mixing 18.61 g of EDTA disodium salt (Sigma Aldrich E4884), 6.81 g of sodium borate decahydrate (Sigma Aldrich S9640), 30 g of sodium lauryl sulfate (Sigma Aldrich L4509), 10 ml of triethylene glycol (Sigma Aldrich 95,126), 4.56 g of sodium acid phosphate (Sigma Aldrich S3139) in 1000 ml of distilled water. The sample‐enzyme mixture was boiled for 1 hr, filtered and then dried at 100°C for 5 hr. After drying, the crucible containing the solid residue was placed in a muffle (5 hr, 525°C). The NDF was reported as percentage.

The carbohydrate percentage was calculated by the difference between the previous percentages. All these analyses were performed in triplicate.

### 2.4 Carbohydrate analyses

#### 2.4.1 Total and reducing sugars

The total sugar content was determined using the Phenol‐Sulfuric methodology (Dubois et al., 1956). Briefly, 1 ml of sample was mixed (in dark conditions) with 0.6 ml of 5% phenol (Sigma‐Aldrich 33517) using a vortex (Barnstead Thermolyne Maxi‐Mix ii). Subsequently, 3.6 ml of concentrated H_2_SO_4_ (Merck 100,731) was added to the tubes, shaking, and allowing to stand for 30 min. At the end of this period, they were placed in a cold‐water bath to stop the reaction. The sample was read in a spectrophotometer (Thermo Scientific‐Genesys Uv) at a wavelength of 490 nm. The concentration of total sugars was determined through a glucose calibration curve (5–60 µg/ml).

The quantification of reducing sugars was carried out with the 3.5 dinitrosalicylic acid methodology (DNS) (Miller, 1959); DNS was prepared by dissolving 0.8 g of NaOH (JT Baker 1310‐73‐2) in water and mixing with 15 g of tetrahydrate sodium‐potassium tartrate (JT Baker 6381‐59‐5) and 0.5 g of DNS (3.5‐dinitrosalysilic acid, Sigma Aldrich D0550), making up the solution to 50 ml in a volumetric flask. A glucose calibration curve (50–500 µg/ml) was prepared. The DNS reagent (1 ml) was added to the sample, and it was placed in boiling water for 10 min. It was allowed to stand at room temperature for 15 min, and the absorbance was read in a spectrophotometer (Thermo Scientific‐Genesys Uv) at a wavelength of 540 nm. The concentration of reducing sugars was determined by interpolation of the absorbance reading in the calibration curve.

#### 2.4.2 Starch and fiber evaluation

##### Total starch

The percentage of total starch was determined using the Megazyme K‐TSTA kit (Megazyme International Ireland Ltd.). The samples were weighed (100 mg), 0.2 ml of 80% ethanol added and homogenized in a vortex (Barnstead Thermolyne Maxi‐Mix ii). Immediately, 3 ml of thermostable α‐amylase was added, heating to the boiling point, and stirring every 2 min for a total of 6 min, and then cooling in a water bath until reaching 50°C. Amyloglucosidase (0.1 ml) was added, vortexing and incubating for 30 min. The enzymatic hydrolyzed sample solution was poured into a 100 ml volumetric flask, and an aliquot of 25 ml was taken and centrifuged (Dynamic Velocity 18R, Metrix^®^) at 3420 ×* g* for 10 min, extracting 0.1 ml of the supernatant (by duplicate) to continue the analysis. Simultaneously, a control sample (blank) was run from the beginning of the analysis, and a glucose standard was run starting at this point. The four tubes (sample duplicates, blank, glucose standard) were added with glucose oxidase/peroxidase solution (3 ml) and placed in a water bath at 50 ºC for 20 min. Finally, the absorbance of the sample was read at 510 nm (Genesys 10UV Scanning, ThermoScientific, USA). The percentage of total starch was obtained applying Equation (1),

(1) $$\%\;\text{total}\;\text{starch}=\Delta A\times \frac{F}{W}\times VF\times 0.9$$

where ΔA is the sample absorbance versus blank absorbance, W is the weight of the sample in milligrams, F is the factor (100 μg of glucose standard / Absorbance of glucose standard), and FV is the final volume (100 ml).

##### Total dietary fiber

Total dietary fiber (TDF) was determined using a Sigma‐Aldrich kit (Sigma Aldrich, TDF‐100A). Samples were weighed in quadruplicates (0.5 g‐W1) and placed in beakers; then 50 ml of phosphate buffer (pH 6) and 50 µl of thermostable α‐amylase (pH 6.0‐Sigma Aldrich A3306) were added, heating the mixture at 95°C for 15 min. The sample‐amylase solution was cooled to room temperature, and its pH adjusted to 7.5 by adding 5 ml of 0.275 N NaOH solution. A recently prepared 50 mg/ml protease solution (Sigma Aldrich P3910) in phosphate buffer (pH 7.5) was prepared and 50 µl of the enzyme solution was poured into each beaker, covering with an aluminum foil, heating at 60°C for 30 min, and allowing the reaction to cool down to room temperature. The pH was adjusted to 4 (0.325 M HCl, 10 ml), and 50 µl of amyloglucosidase (Sigma Aldrich A9913) was added, incubating the sample for 30 min at 60°C. Ethanol 96% (260 ml) was added and left overnight at room temperature to allow for total precipitation to occur. The sediment was quantitatively recovered in a celite (0.25 mg) prepared crucible by filtering the solution. The residues in the beaker were washed with three portions of 10 ml of 78% ethanol, two portions of 5 ml of 96% ethanol, and two portions of 5 ml acetone. The crucibles were left overnight in an oven at 105°C, allowed to cool in a desiccator, and weighed (W2). Two crucibles were taken for the determination of protein (nitrogen Kjeldahl‐6.25), while the remaining crucibles were incinerated for 5 h at 525°C, allowed to cool in a desiccator, and the weight (W3) was taken to determine the ash weight. Total dietary fiber content (TDF) was calculated as follows.

(2) $$\text{Residualweight}=W_{2}‐W_{1}$$

(3) $$\text{Ash}\;\text{weight}=W_{3}‐W_{1}$$

(4) $$\%TDF=\left[\frac{R_{\mathrm{sample}}‐P_{\mathrm{sample}}‐A_{\mathrm{sample}}‐B}{\mathrm{SW}}\right]*100$$

where R is the average weight of the residue (mg), P is the mean protein weight (mg), A is the average ash weight (mg), SW is the average sample weight (mg), W1 is the Celite + crucible weight, W2 is the Residue + Celite + crucible weight and W3 is the ash + celite + crucible weight.

### 2.5 Identification of sugars by HPLC

#### 2.5.1 Sample preparation

Dry sample (0.5 g) was suspended in 40 ml of hot water (60°C, 6.5 < pH < 8). After 2 min of manual mixing, the solution was brought to a final volume of 50 ml. The pH was controlled in each step to maintain the solution within the desired value range (6.5–8). The sample was then centrifuged at 1,008 × *g* for 10 min (Metrix Lab Dynamica). After centrifugation, an aliquot of the supernatant (1.5 ml) was filtered through a 0.45‐µm acrodisc nylon membrane (Millex^®^) and then through 0.2‐µm nylon filters (Millex^®^).

#### 2.5.2 Standard preparation

A kit of monosaccharides consisting of D (−)Fructose, D (+)Glucose, (Sigma‐Aldrich, 47267), as well as a solution of D‐(+)‐galacturonic acid monohydrated (Sigma‐Aldrich, 48280‐5G‐F, ≥97.0%), sucrose (Sigma‐Aldrich, 47289) and inulin from chicory (Sigma‐Aldrich, l2255‐10G), at a concentration of 10 mg/ml in water, was used. Before analyses, standards were filtered through 0.2‐µm nylon filters (Millex).

#### 2.5.3 Chromatography

Chromatographic equipment consisted of a 1200 Infinity System quaternary pump (Agilent); data were obtained from the refractive index detector (RID, 1260, Agilent). An Agilent Hi‐Plex H column (7.7 × 300 mm, 8 µm, PL1170‐6830) was used for the determination of pectin (galacturonic acid), and a Hamilton Ca column (7.8 × 305 mm, 8 µm, 79436) was used for the detection of inulin, sucrose, glucose, and fructose. The chromatographic separation of galacturonic acid was performed with 0.01 M H_2_SO_4_ as a mobile phase, at a 0.4 ml/min flow rate, 20 µl injection volume, and a column and detector temperature of 55°C. The detection of inulin, nystose, sucrose, glucose, and fructose was carried out with water as a mobile phase, at 0.4 ml/min flow rate, 20 µl injection volume, column temperature of 85°C, and detector temperature of 55°C.

### 2.6 In vitro digestion of starch (glycemic index)

The glycemic index was measured following the methods of Salgado‐Cruz et al. (2017), and Goñi et al. (1997) with some modifications. The jicama samples were weighed (10 mg), mixed with 2 ml of HCl‐KCl buffer (pH 1.5), and added with 0.04 ml of pepsin (1 g of pepsin, Merk 107185/10 ml of HCl‐KCl buffer). Samples were kept in an incubator shaker (Barnstead International‐SHKA4000‐7 IOWA, EUA) at 40ºC for 1 h.

After incubation, 5 ml of tris‐maleate buffer (pH 6.9) was poured to adjust pH (6.75–7.10) of the sample solution followed by the addition of 1 ml of tris‐maleate buffer containing 2.6 U of α‐amylase from porcine pancreas (A −3176, Sigma‐Aldrich Inc.); the sample was incubated at 37°C. Aliquots of 0.5 ml were taken every 30 min from 0 to 180 min. To inactivate the enzyme (α‐amylase), these aliquots were heated to 100°C and kept at this temperature for 5 min while shaking. Then, 0.6 ml of 0.4 M sodium acetate buffer (pH 4.75) and 12 µl of *Aspergillus niger* amyloglucosidase (A7420, Sigma‐Aldrich Inc.) were added; these samples were incubated at 60°C for 45 min.

Finally, glucose concentration was measured with the glucose oxidase‐peroxidase kit (GAGO20, Sigma‐Aldrich Inc.). The kinetics of in vitro starch digestion was followed by a nonlinear model. The results were multiplied by 0.9 (stoichiometric glucose/starch conversion factor) to convert glucose to starch, as Salgado‐Cruz et al. (2017) reported. The glycemic index was calculated using the area under hydrolysis curve (AUC), which was obtained by fitting the experimental data to a two‐parameter exponential model. The (Equation 5) applied was:

(5) $$C=C_{\propto }\left(1‐e^{‐kt}\right)$$

where C is the percentage of hydrolyzed starch at time t. C_∝_ is the percentage at equilibrium of hydrolyzed starch, normally after 180 min, k is the kinetic constant, and t is the time (min). The parameters C_∝_ and k were estimated using the software SIGMAPLOT version 11 for MS Office and an exponential model was used to calculate AUC (Equation 6):

(6) $$AUC=C_{\propto }\left(t_{f}‐t_{0}\right)‐\left(\frac{c_{\propto }}{k}\right)\left[1‐exp‐k\left(t_{f}‐t_{0}\right)\right]$$

where, “t_f_” is the final time (180 min), “t_0_” is the initial time (0 min). The hydrolysis index (HI) was expressed as the ratio of the area under the hydrolysis curve (AUC) of the sample to the AUC of the white fresh bread. Then, the estimated glycemic index (eGI) was calculated by using the equation reported by Granfeldt et al. (1992).

(7) $$eGI=8.189+\left(0.862*HI\right)$$

### 2.7 Statistical analysis

Data are presented as the mean ± *SD*. Statistical analyses were performed using Sigma‐Plot software (v.14, SYSTAT) with a probability value *p* <.05 considered significant. The differences between the groups were assessed using ANOVA‐Tukey's test. Also, a Pearson correlation text was carried out, using the same software.

## 3 RESULTS AND DISCUSSION

### 3.1 Root diameter and growing rate

**FIGURE 2:**
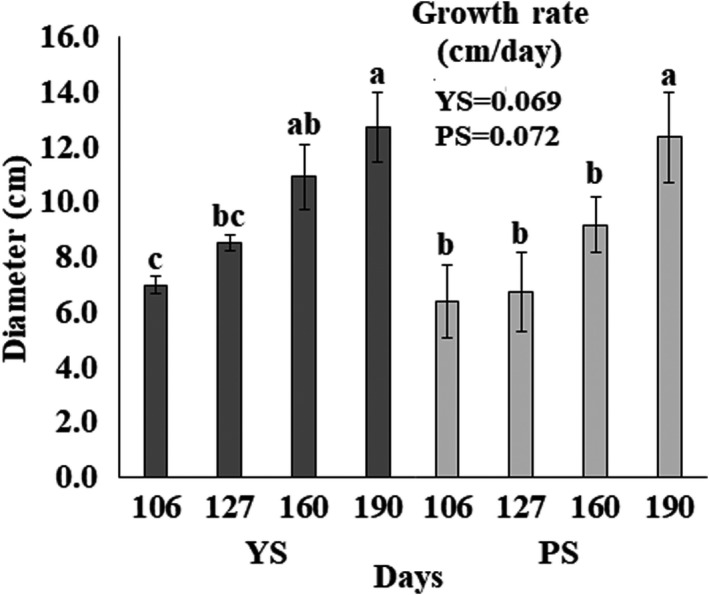
Changes in jicama's diameter during development. YS: Yellow seed; PS: Purple seed. The growth rate was evaluated from the slope of the diameter – days curve. Values with different letters in each group (YS or PS) are significant different (*p* <.05)

The tuberous roots harvested at different times (106, 127, 160, 190 days) presented different diameters, ranging from 6.7 to 12.7 cm for YS and from 6.39 to 12.33 cm for PS (Figure 2).

Figure 2 shows that root diameters change during maturation and as expected, their size increased as time passed. Based on the statistical analysis, it can be said that development of the root between both materials (YS and PS) was similar as they reached the same diameters (*p* >.05) at the end of the maturation period. The development of roots has been explained by different authors (Chaweewan & Taylor, 2015; Mehdi et al., 2019; Wang et al., 2016; Zierer et al., 2021), citing that during growing, the root swells, the expansion of central xylem pushes the cells into the intact endodermis and, as time goes on, the enlarged roots, formed almost exclusively by parenchymal xylem cells, accumulate starch. It has also been reported that roots almost double in size (measure as root diameter) during a 16‐week period of growth (Fernandez et al., 1997), behavior that is similar to the one observed in our work.

### 3.2 Changes in jicama chemical composition

**FIGURE 3:**
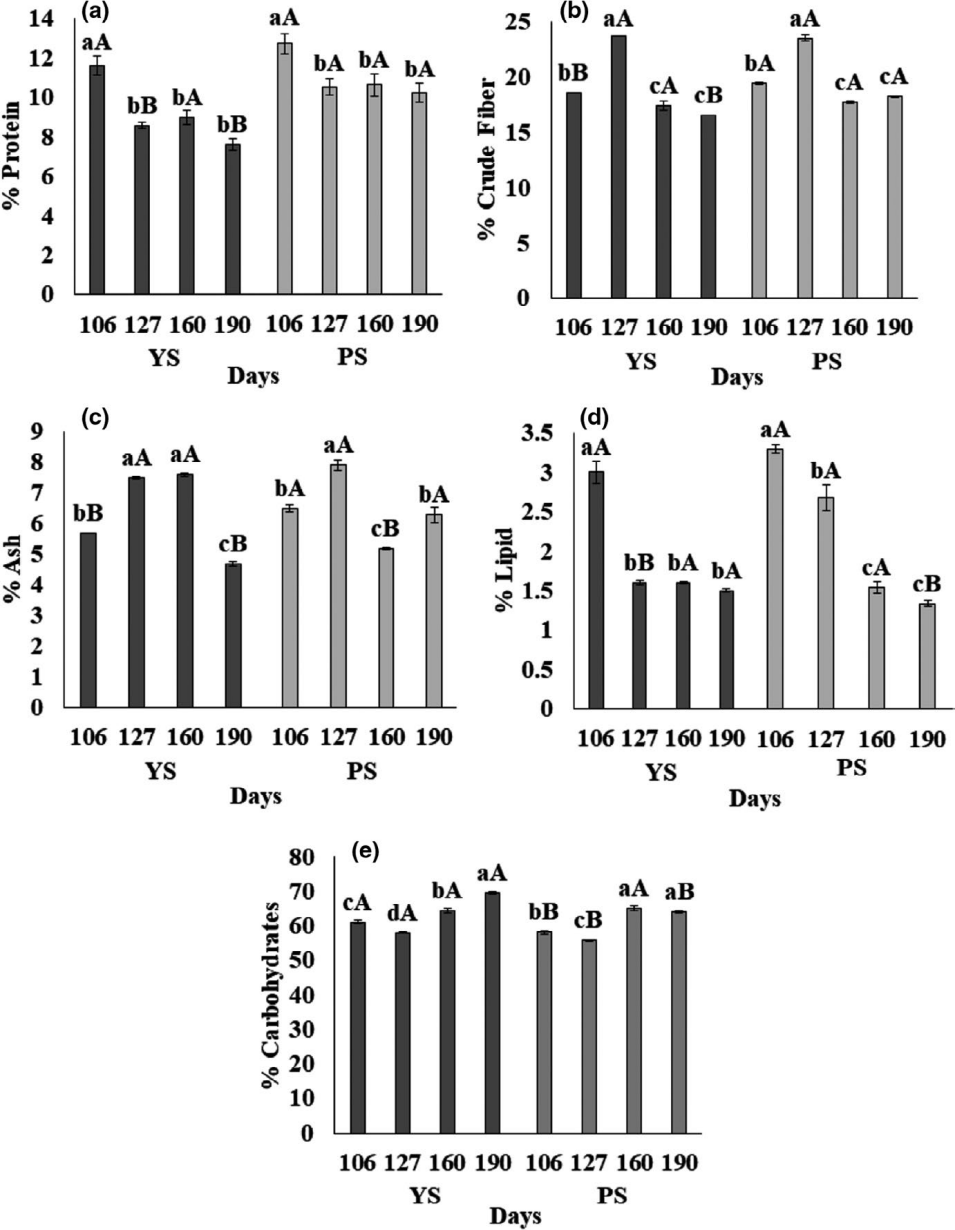
Proximate analysis results (%, db‐ dry base) of the two jicama materials at different stages of growth; YS: Yellow seed; PS: Purple seed. (a) % Protein, (b) % Crude Fiber, (c) % Ash, (d) % Lipid and (e) % Carbohydrates. Bars with different small letters are significantly different inside the group (YS or PS). Bars with different capital letters are significantly different between the same developmental stage

The changes in the chemical composition of the two jicama materials at different stages of growth are presented in Figure 3a–e.

When analyzing the protein content (Figure 3a), it decreases from 11.6 ± 0.48 to 7.61% ± 0.29% for YS and from 12.7 ± 0.51 to 10.5% ± 0.43% for PS up to 127 days of growing, remaining statistically invariable (*p* >.05) for both samples for the rest of the maturing period. It can also be observed from these results that the PS path follows the same behavior as YS. This behavior agrees with that reported by Nursandi et al. (2015), where the protein content decreased over time until reaching commercial maturity. The protein values cited by Nursandi et al. (2015) are lower than those obtained in our research. These differences are related to the seed's genotype and the tuber development stage. The decrement of protein up to a constant value reached at 127 days for both materials, could be related to the protein function in the root. Forsyth and Shewry (2002) published that *Pachyrhizus* is a perennial crop, in which the tuberous roots act as storage organs for carbohydrates rather than as propagules, showing a strictly controlled low protein accumulation adjusted to the availability of resources (Fernie & Willmitzer, 2001; Shalit et al., 2009).

In Figure 3‐b, the crude fiber contents had their highest significant values at 127 days in both materials, decreasing thereafter. When comparing the YS and PS materials at the same maturity stage, no statistically significant difference (*p* =.05) was found for this component at most of the development stages. For YS, the values ranged from 18.6 ± 0.01 to 16.6% ± 0.02% and for PS from 19.4 ± 0.11 to 18.2% ± 0.06%. These results can be compared with those reported for yacón at different growing times, varying from 13.38% to 28.49% (Silva et al., 2018). Huang et al. (2007) reported that the crude fiber content is higher in “immature” tubers than in “mature” ones. This situation can be related to the early stages of root development, where the xylem and phloem start growing and conclude with the formation of the cell wall structure.

The ash levels for YS were 5.7% ± 0.001% at the beginning of growth, and during development, their concentrations were stable from 127 to 160 days; despite this, in the end, a decrease was observed (4.7% ± 0.09%). Concerning PS, this material did not present a homogeneous content, as shown in Figure 3c; PS at 106 days has a level of 6.5% ± 0.11%, and at 190 days, a value of 6.3% ± 0.25%. This behavior had already been reported by Tréche and Agbor‐Egbe (1996) in two varieties of yam, where the ash content did not show uniform or orderly changes with growth. The changes in the ash content during tuberous root development can be related to pH conditions, root variety, water supply, climate, and seasonal variations, as well as to agronomic practices. The ashes indicate the minerals available for proper root growth (Leterme et al., 2006).

Regarding lipids (Figure 3d), a decrease during root growth was observed for both materials; for YS, it ranged from 3.02% ± 0.14% to 1.5% ± 0.02% and in PS from 3.2% ± 0.05% to 1.3% ± 0.04%. This observation is similar to those reported during the potato tuberization process (Berkeley & Galliard, 1974). Lipids are precursors of phytohormones that are responsible for cell division, formation, and enlargement at the beginning of the tuberization process. It is expected that these parameters will decrease until they reach their full development and continue with the widening of the root (storage of starch) (Sarkar, 2008).

Carbohydrate content (Figure 3e) showed a slight increase during root growth for both materials, with a variation of 61.1% ± 0.33% to 69.5% ± 0.38% and 58.2% ± 0.47% to 64% ± 0.32%, respectively, being the most notable change, for both samples, after 127 days of growing. The tuberous roots are storage organs, and carbohydrates play a fundamental role during the tuberization process, since as the root grows, they increase to satisfy the different physiological requirements of the plant. In this sense, Gregory and Wojciechowski (2020) and Cérantola et al. (2004) reported that sucrose is one of the main inducers for root development in the synthesis of different polysaccharides. Additionally, during the tuberization process, a massive accumulation of starch occurs in the tuber, which represents a high demand for carbohydrates, requiring the presence of sugars such as glucose and fructose (Morales et al., 2018) for their synthesis.

The functional relationship between these mechanisms intervenes in the productivity and quality of the tubers. Any stress that negatively affects these processes can mainly inhibit tuberization and tuber growth (Dahal et al., 2019). The principal abiotic stresses, such as high temperatures, drought, soil salinity, and nutrient stresses, negatively affect these processes and substantially reduce plant growth, tuberization and increase in the volume of tubers and, therefore, the yield and quality of tubers (Minhas, 2012; Wang‐Pruski & Schofield, 2012).

However, the loss of yield due to stress depends on the plant's age, severity of stress, and growth stage. Early stress is more detrimental to tuberization, increased volume and yield of tubers due to reduced carbon assimilation rate and decreased assimilated partition in tubers (Dahal et al., 2019).

### 3.3 Carbohydrate analyses: Reducing and non‐reducing sugars, soluble fiber, and starch

**FIGURE 4:**
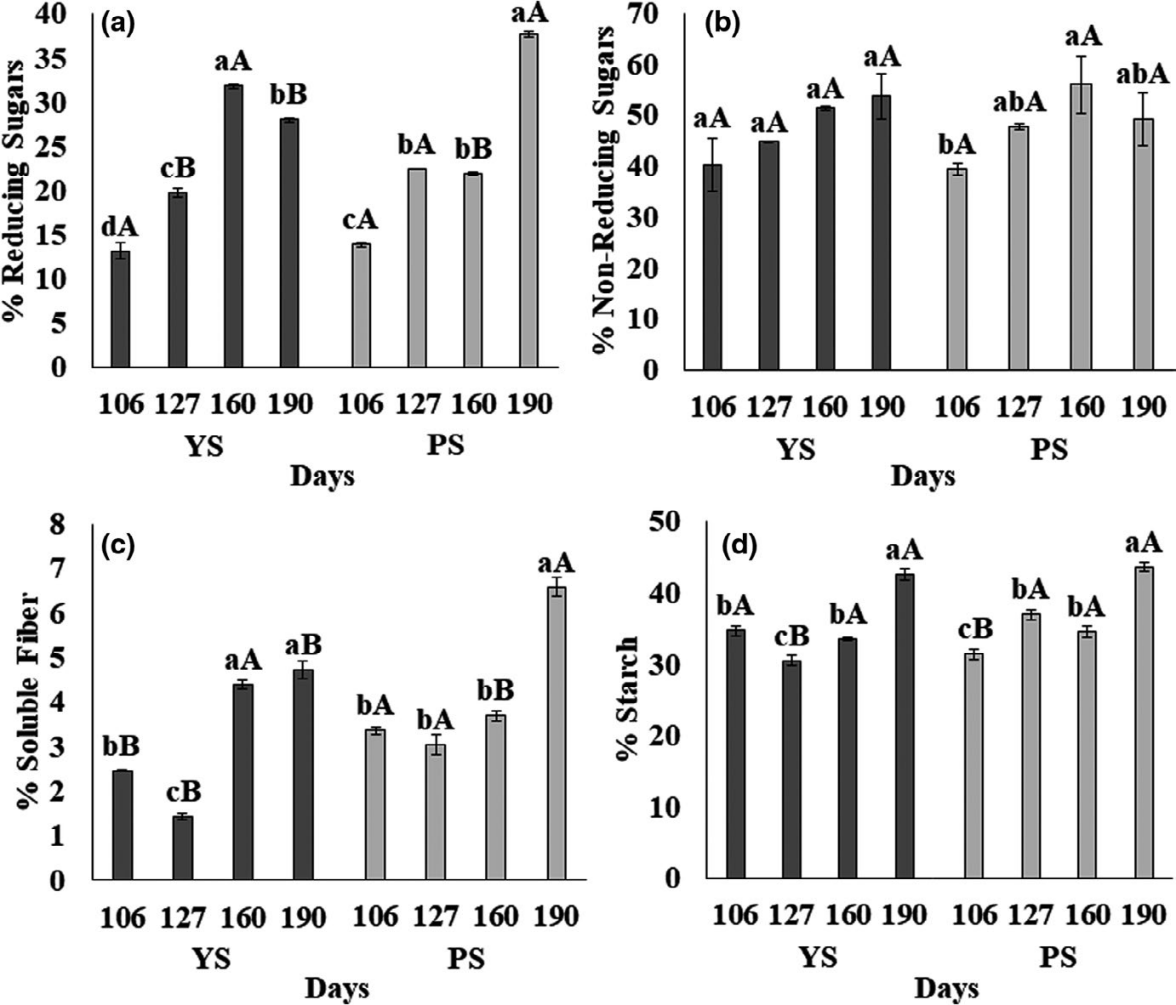
Carbohydrate composition (% db‐ dry base) of two jicama materials (YS and PS) in different growing stages; YS: Yellow seed; PS: Purple seed; (a) % Reducing Sugar; (b) % Nonreducing Sugar; (c) % Soluble Fiber; (d) % Starch. Bars with different small letters are significantly different inside the group (YS or PS)

The values obtained for sugars (reducing, non‐reducing), soluble fiber, and starch in this study are observed in Figure 4. It is noticed that carbohydrates increased in concentration during the different harvest stages.

Regarding reducing sugars (Figure 4a), YS showed differences during root development with a 13.2% ± 0.9% content at the beginning of tuberization and 28.0% ± 0.2% at the end of the period; for PS, 13.9% ± 0.2% was recorded at the beginning of tuberization and 37.9% ± 0.3% at the end. Regarding nonreducing sugars, all the roots had values higher than 30%, with few changes during development (*p* >.05) and with no statistical differences between the samples (YS vs. PS). This same behavior has been reported for the ahipa species by Leonel et al. (2005) and even in other tubers, such as potatoes. This behavior can be attributed to the inhibition of the enzymatic hydrolysis of sucrose, which is the principal nonreducing sugar, during the development of tuberization. As the root grows and the foliage dies (during harvest, induced or naturally), the enzymatic activity converts the nonreducing sugars into reducing sugars, maintaining the levels throughout root development (Sowokinos, 2007).

Soluble fiber (Figure 4c) content increased during root growth, peaking at 160 days of development for YS (4.72% ± 0.10%) and at 190 for PS (6.56% ± 0.22% YS). This change may be related to the fact that part of the soluble fiber is composed of cell wall polysaccharides, such as pectic polysaccharides, where its presence indicates that the wall development cycle has been completed (Wennberg et al., 2002). Tréche and Agbor‐Egbe (1996) and Afoakwa and Sefa‐Dedeh (2001) reported a similar pathway in yam species.

Concerning starch, YS samples showed a content of 34.8% ± 0.74% at the beginning of tuberization (Figure 4d), which reached 42.7% ± 0.80% at 190 days. PS samples followed a similar path presenting the lowest starch content at 106 days (31.4% ± 0.69%), reaching the highest value at 190 days (43.7% ± 0.65%). Similar behavior was reported for cassava roots (Wang et al., 2016), where the highest accumulation of starch occurred at the end of development. These results are related to plant growth, during which starch accumulates in the leaves through the day, degrading in the same way at night; this pattern is modified by a wide range of parameters, such as the magnitude of irradiation, length of days, CO_2_ concentration, and water supply (Stitt & Zeeman, 2012). From these results, it is clear that the main carbohydrate in jicama solids is starch, followed by fiber, where soluble fiber represents less than 10%.

### 3.4 Identification of sugars by HPLC

**FIGURE 5:**
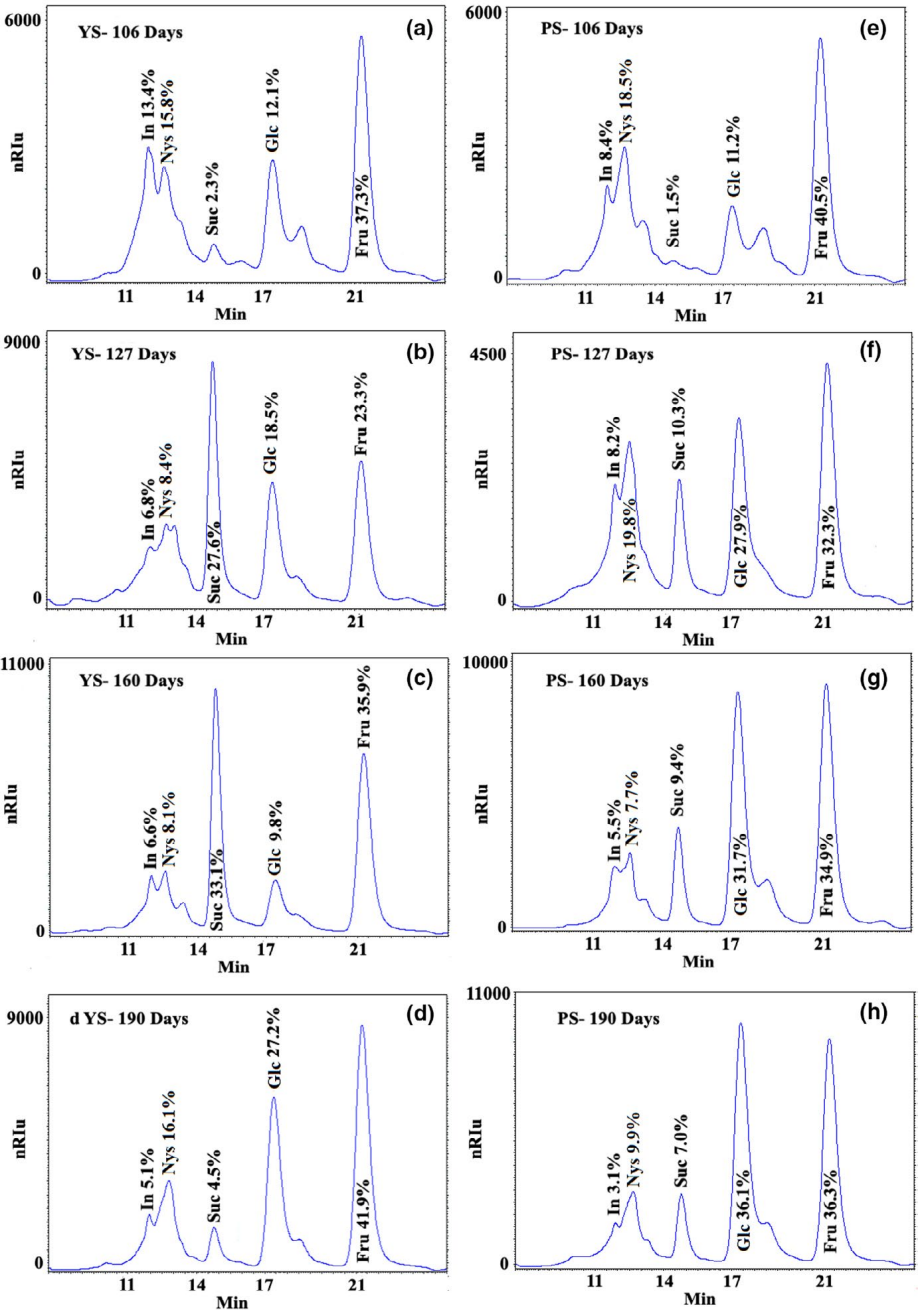
HPLC chromatograms of two jicama materials (YS and PS) evaluated at different growing stages. YS: Yellow seed; PS: Purple seed. Retention times: inulin (In): 11.9 ± 0.05 min; nystose (Nys): 12.7 ± 0.08, sucrose (Suc): 14.8 ± 0.02 min; glucose (Glc): 17.4 ± 0.01 min; fructose (Fru): 21.3 ± 0.01 min; Determination in Hamilton Ca^+2^ column

**FIGURE 6:**
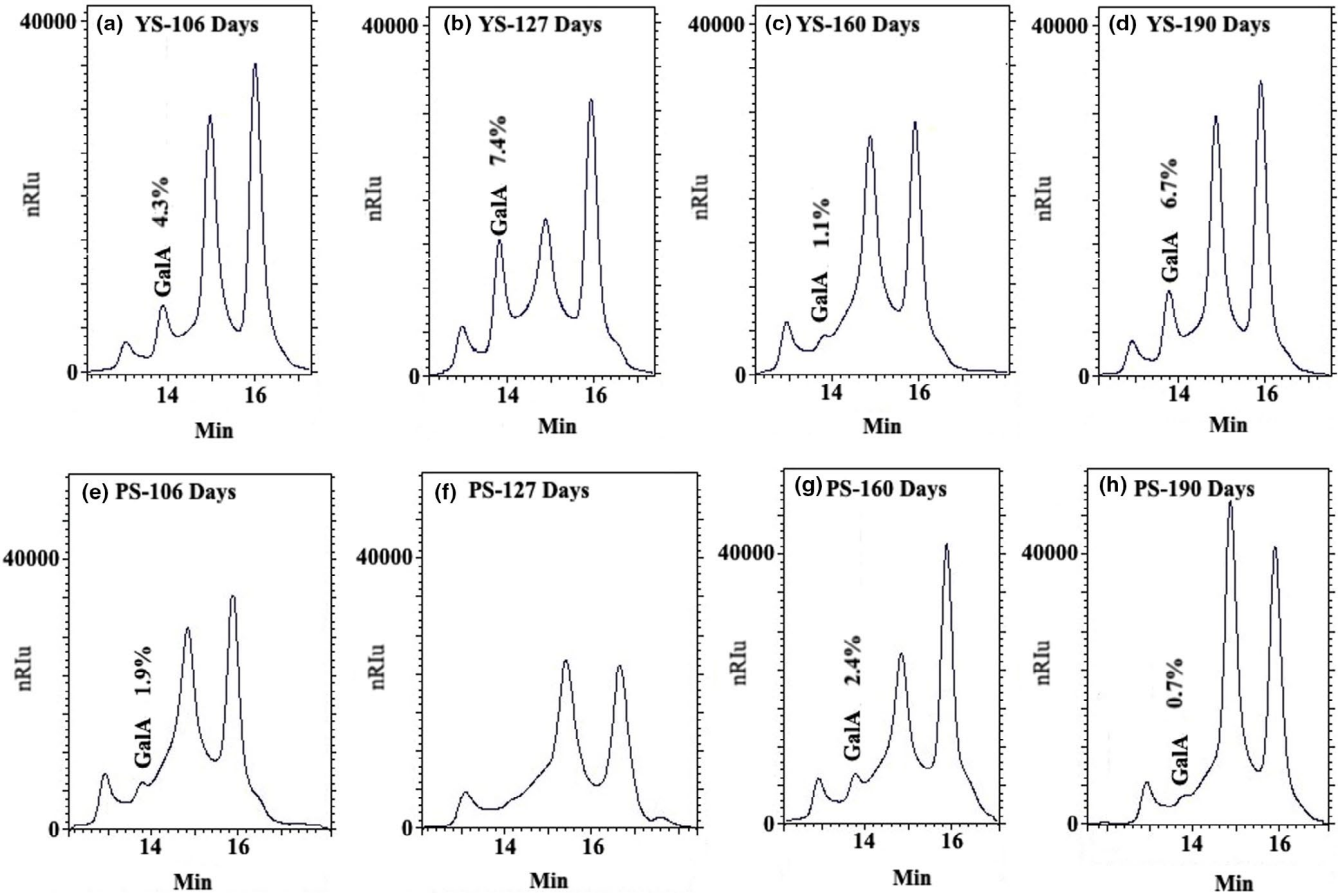
Galacturonic acid (GalA) detection by HPLC (RT: 14.1 ± 0.3 min) on two jicama materials (YS and PS) at different growing stages. YS: Yellow seed; PS: Purple seed; RT: retention time. Determination on Agilent HI‐PLEX‐H column

Jicama samples were analyzed by HPLC, confirming the presence of fructooligosaccharides, sucrose, glucose, and fructose (Figure 5), as well as pectin (Figure 6).

The highest proportion of inulin was detected at the beginning of the root sampling (106 days), observing in this period a maximum of 13.4% ± 0.69% for YS and 8.4% ± 0.18% for PS. The lowest inulin values (5.1% ± 0.10% and 3.1% ± 0.09%) were noticed in both samples (YS and PS, respectively) at 190 days, having almost half of the maximum content. YS showed higher levels of inulin than PS most of the time. This behavior has also been reported in artichoke and in cultivated and wild thistle (Mellido & Raccuia, 2007).

Inulin is used as a reserve carbohydrate in plants for regrowth after defoliation and germination in Spring. It is also related to plant protection during drought or cold seasons and when growing in saline soils (Mellido & Raccuia, 2007).

Nystose, a linear fructooligosaccharide made up of one glucose and three fructose molecules (Mingruo, 2009), was also identified in the samples. The highest percentage for YS was observed at the end of root development (190 days 16.1% ± 0.1%), while for PS at 127 days (19.8%), without having, any of the materials, a homogeneous behavior. These types of fluctuations have been reported for yacon (*Smallanthus sonchifolius*) and burdock root (*Arctium lappa L*) (Imahori et al., 2010; Lim, 2016b) and explained as the hydrolysis of an excess of sucrose produced during photosynthesis (Mohammadi et al., 2021). Once sucrose reaches a specific threshold, fructans are synthesized by the activity of fucosyltransferase (Mohammadi et al., 2021), and stored in sinking organs; during the flowering stage, these carbohydrates are degraded, and sucrose is transported to flowers as new sink organs (Gupta & Kaur, 2000). Fructooligosaccharides degradation and fructose release result in low DP (degree of polymerization) fructooligosaccharides (Van Laere & Van den Ende, 2002), information that is in accordance with our results, as inulin and nystose decrease.

This is an important finding since the fructooligosaccharides present as part of jicama components, although mentioned in some reports, had not been chemically or chromatography analyzed. Fructooligosaccharides have attracted attention in the food industry due to their beneficial effect on the health of those who consume them, as they are considered a prebiotic (Romero et al., 2015).

The proportion of sucrose (Figure 5) was different in all growth stages, with significant differences in the development of the roots in both samples, with YS showing the highest values most of the time, reaching 33% at 160 days. It has been reported that sucrose is an essential component in the tuberization process, and any stressing condition (temperature, stress, climate, and water) promotes a decrease in this component, resulting in massive rearrangements of tuber metabolism (Fernie & Willmitzer, 2001). It has also been cited that when measuring sucrose in different cassava tissues, its content did not show uniform or ordered changes with growth, possibly because it could be dynamically interconverted into starch (Li et al., 2016) or another plant storage component, as it is the primary sugar transported in the phloem of most plants (Stein & Granot, 2019).

Glucose content (Figure 5) showed significant differences throughout root growth, increasing in concentration from 12.1% ± 1.0% to 27.2% ± 0.04% for YS and from 11.2% ± 0.04% to 36.1% ± 0.44% for PS, while fructose percentage between materials showed fewer variations. The fructose content for all samples was higher (23.3% ± 1.08% to 41.9% ± 0.11% for YS; 32.3% ± 0.06% to 40.5% ± 0.77% for PS) than glucose. This result could be related to fructooligosaccharide hydrolysis into fructans with a lower degree of polymerization during root development, rendering sucrose and fructose (Baert, 1997). Furthermore, it is essential to mention that in storage tissues that actively synthesize starch, most of the incoming sucrose is divided by sucrose synthase into UDP‐glucose and fructose, and another portion by invertase to produce glucose and fructose. UDP‐glucose can be integrated into the synthesis of starch (and structural polysaccharides). Additionally, developing potato tubers possess an efficient pathway for utilizing fructose as a starch precursor. The preferential action of hexokinase toward fructose compared to glucose may provide an important checkpoint for the rapid turnover of fructose derived from the action of sucrose synthase and explains the high glucose: fructose ratio that is found in tubers in development (Davies & Oparka, 1985).

Another component seldom reported for jicama and detected in the YS and PS roots in a small proportion was galacturonic acid (Figure 6), which is kindred to pectin, along with xylose and arabinose, sugars not evaluated in this work, but possibly present in Figure 5 (peak between 17 and 21 min) and overlap by glucose and fructose in Figure 6 (elution time ≈16 min) as has been reported in Hamilton (2021).

The pectin behavior may be possibly related to the fact that pectic polysaccharides within the fiber elongation period increase in the initial stage of development and decrease throughout the cell wall thickening stage (Tokumoto et al., 2002). Besides, in jicama, the presence of pectinolytic enzymes (i.e., polygalacturonase, pectinases, and rhamnogalacturonan) has been reported (Pressey, 1993; Ramos de la Peña et al., 2013).

It is essential to mention that dietary pectin has various biological activities related to the reduction of lipid, insulin, glucose levels, and immunomodulatory effects by increasing the levels of the proinflammatory cytokines IL‐17, IFN‐γ, and TNF‐α levels (Merheb et al., 2019). Furthermore, pectin forms a physical barrier that protects the epithelium against opportunistic microbial invasion during stress (Wikiera et al., 2014).

From these chemical composition results, the aforementioned nutraceutical properties cited by different authors, and specifically the hypoglycemic effect, can be considered to be related to the presence of pectin and fructooligosaccharides, as well as to the native starch, acting as fiber (Bertolini, 2010; Kendall et al., 2010), which must give to the jicama a low glycemic index.

### 3.5 Estimation of the glycemic index

**FIGURE 7:**
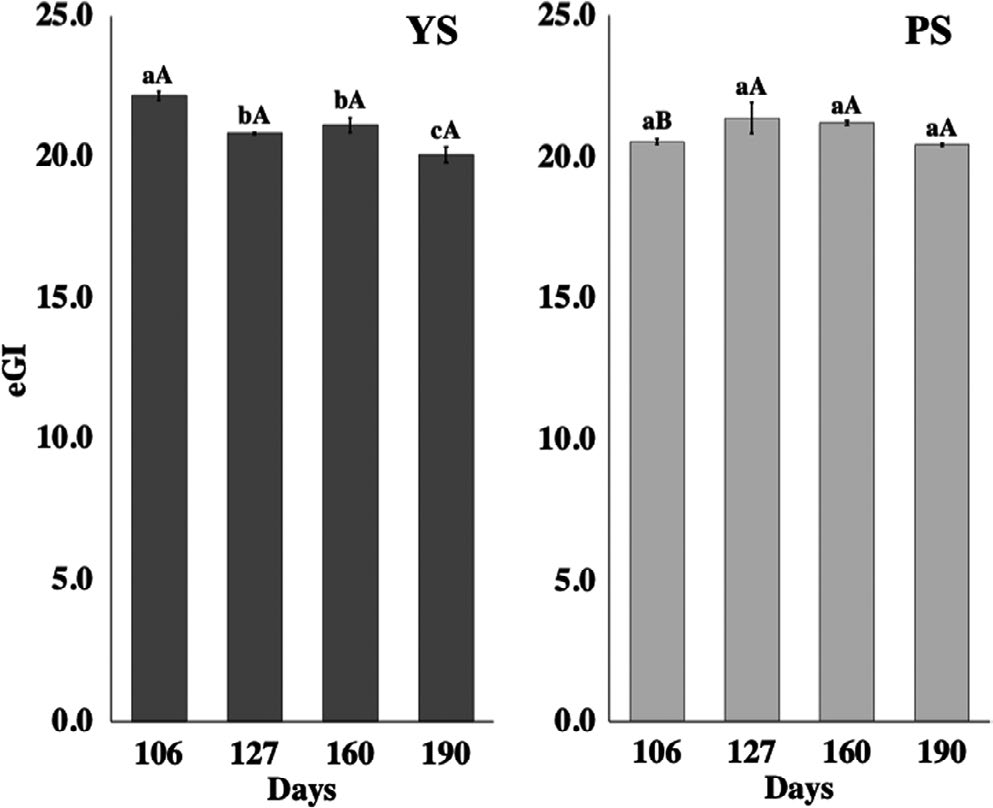
Estimated glycemic index (eIG) in jicama during different growing stages. The mean value ±*SD* is presented. Bars with different small letters are significantly different inside the group (YS or PS). Bars with different capital letters are significantly different between the same developmental stage

The GI values for YS and PS samples are presented in Figure 7.

The yellow seed variant showed a statistically significant difference (*p* <.05) during the maturation times, where the highest value was observed at 106 days (eGI = 22.2), with a tendency to decrease through development stages (190 days, IG = 20.1). PS did not show a statistically significant difference (*p* >.05) in its GI values during root growth. When comparing materials, the GI between the jicama samples (YS and PS) was only statistically different (*p* <.05) for YS at 106 days of growth. However, all measurements were very close to each other. This behavior agrees with the proportion of nutraceutical polysaccharides (fructooligosaccharides, pectin, native starch) found in the jicama samples, and in their maturation stages. In this regard, Jenkins et al. (1981) expressed that the GI of foods is mostly related to the nature of the starch and the amount of fiber, among others (Thorne et al., 1983). In other works, Bertolini (2010) and Douzals et al. (1996) cited that the native starch is less susceptible to the enzymatic action, and since jicama is mostly consumed as a raw product, its starch remains in its native form, even during the digestion process.

Based on our results, jicama can be classified as a low glycemic index (GI) food, taking into consideration the Atkinson et al. (2008) GI classification report, which cites that those foods with GI values lower than or equal to 55 are classified as low. In addition, they also described the GI of fried or cooked tuberous roots with GI values between 87 and 53.

It is important to mention that the consumption of low GI foods like jicama minimizes the rate of glucose absorption, which in turn decreases inulin production and circulating lipids. This results in a low postprandial glucose response, as glucose absorption in the small intestine is reduced, leading to better glycemic control (Kendall et al., 2010). This makes jicama a potential functional food. However, more studies are needed to understand the carbohydrate digestion process in jicama.

Finally, through a Pearson's correlation, the root development (days) was correlated for YS with root diameter (R = 0.999, *p* =.001) and nonreducing sugars (R = 0.982, *p* =.0185), which could be associated with the production of starch. Additionally, nystose and sucrose showed a high correlation (R = 0.999, *p* =.01), with the value of nystose decreasing at higher sucrose concentrations. PS was correlated with diameter (R = 0.973, *p* =.026) and inulin (R = −0.978, *p* =.02), where the larger the root, the lower the inulin concentration. This could mean that inulin is used in the early stages of development as an energetically cheaper way to have initial reserve materials before giving way to starch build‐up.

## 4 CONCLUSIONS

The jicama carbohydrates of nutraceutical interest are related to the state of maturity of the root, being inulin the first to be used, decreasing their content to almost 39% of its initial value during development and generating nystose, a fructooligosaccharide of lower DP (degree of polymerization). Starch content increased throughout the whole period, varying from 34% to 43% in both materials (YS, PS). The chemical composition was related to the jicama variety, having PS the higher sucrose final content (1.5%–7.0%) and the lower final pectin (galacturonic acid) concentration (1.9%–0.7%). Based on simple sugar (fructose, glucose and sucrose) proportions, the sweeter product was YS at 160 days. These results provide extensive knowledge about jicama since it was possible to identify fructooligosaccharides, pectin, and sucrose and observe the changes in carbohydrates composition during the tuberization. Furthermore, this research shows that jicama is a food with a low glycemic index; this information will help establish the right time to harvest and the benefits of jicama. More studies are needed in this area.

## CONFLICT OF INTEREST

The authors declare that there is no conflict of interest.

## AUTHOR CONTRIBUTIONS

**Marcela González‐Vázquez:** Conceptualization (equal); Data curation (equal); Formal analysis (equal); Investigation (equal); Writing – original draft (equal). **Georgina Calderón‐Domínguez:** Conceptualization (equal); Funding acquisition (equal); Investigation (equal); Project administration (equal); Resources (equal); Supervision (equal); Validation (equal); Visualization (equal); Writing – review & editing (equal). **Rosalva Mora‐Escobedo:** Conceptualization (equal); Methodology (equal); Project administration (equal); Resources (equal); Supervision (equal); Validation (equal); Writing – review & editing (equal). **Ma de la Paz Salgado‐Cruz:** Conceptualization (equal); Data curation (equal); Methodology (equal). **José Honorato Arreguín‐Centeno:** Formal analysis (equal); Methodology (equal); Resources (equal). **Ricardo Monterrubio‐López:** Resources (equal); Software (equal).

## ETHICAL APPROVAL

Ethics approval was not required for this research.

## Data Availability

Research data are shared on request.
